# Cover crop functional types differentially alter the content and composition of soil organic carbon in particulate and mineral‐associated fractions

**DOI:** 10.1111/gcb.16296

**Published:** 2022-07-18

**Authors:** Ziliang Zhang, Jason P. Kaye, Brosi A. Bradley, Joseph P. Amsili, Vidya Suseela

**Affiliations:** ^1^ Department of Plant & Environmental Sciences Clemson University Clemson South Carolina USA; ^2^ Department of Ecosystem Science & Management Pennsylvania State University University Park Pennsylvania USA; ^3^ Institute for Sustainability, Energy, and Environment University of Illinois at Urbana‐Champaign Urbana IL USA; ^4^ Section of Soil and Crop Sciences, School of Integrative Plant Science Cornell University Ithaca NY USA

**Keywords:** biomarkers, cover crops, mineral‐associated organic matter, particulate organic matter, plant functional types, soil organic carbon

## Abstract

Cover crops (CCs) can increase soil organic carbon (SOC) sequestration by providing additional OC residues, recruiting beneficial soil microbiota, and improving soil aggregation and structure. The various CC species that belong to distinct plant functional types (PFTs) may differentially impact SOC formation and stabilization. Biogeochemical theory suggests that selection of PFTs with distinct litter quality (C:N ratio) should influence the pathways and magnitude of SOC sequestration. Yet, we lack knowledge on the effect of CCs from different PFTs on the quantity and composition of physiochemical pools of SOC. We sampled soils under monocultures of three CC PFTs (legume [crimson clover]; grass [triticale]; and brassica [canola]) and a mixture of these three species, from a long‐term CC experiment in Pennsylvania, USA. We measured C content in bulk soil and C content and composition in contrasting physical fractions: particulate organic matter, POM; and mineral‐associated organic matter, MAOM. The bulk SOC content was higher in all CC treatments compared to the fallow. Compared to the legume, monocultures of grass and brassica with lower litter quality (wider C:N) had higher proportion of plant‐derived C in POM, indicating selective preservation of complex structural plant compounds. In contrast, soils under legumes had greater accumulation of microbial‐derived C in MAOM. Our results for the first time, revealed that the mixture contributed to a higher concentration of plant‐derived compounds in POM relative to the legume, and a greater accumulation of microbial‐derived C in MAOM compared to monocultures of grass and brassica. Mixtures with all three PFTs can thus increase the short‐ and long‐term SOC persistence balancing the contrasting effects on the chemistries in POM and MAOM imposed by monoculture CC PFTs. Thus, despite different cumulative C inputs in CC treatments from different PFTs, the total SOC stocks did not vary between CC PFTs, rather PFTs impacted whether C accumulated in POM or MAOM fractions. This highlights that CCs of different PFTs may shift the dominant SOC formation pathways (POM vs. MAOM), subsequently impacting short‐ and long‐term SOC stabilization and stocks. Our work provides a strong applied field test of biogeochemical theory linking litter quality to pathways of C accrual in soil.

## Graphical abstract

Plant functional types (PFTs) substantially impact the accrual and persistence of soil organic carbon (SOC) in particulate (POM) and mineral‐associated organic matter (MAOM). We investigated the effect of PFTs (e.g., legume, grass, brassica) on SOC in a long‐term crop cover (CC) experiment. CCs with low litter quality (grasses) accrued higher C in POM, abundant in plant‐derived compounds. However, CCs with high litter quality (legumes) contributed to higher microbial‐derived C in MAOM. Interestingly, mixtures with all PFTs contributed to both POM and MAOM, thus increasing the short‐and long‐term SOC persistence. These results suggest that plant trait‐based understanding of CC practices could improve SOC stocks.

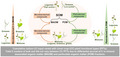

## 1 INTRODUCTION

The sequestration of soil organic carbon (SOC) enhances the soil physical, chemical, and biological health, which is essential for ensuring sustainable food production in a changing climate (Lehmann et al., 2020). As soils are the largest reservoir of terrestrial carbon (C; ~2400 PgC; Batjes, 2000), SOC sequestration is also an important strategy to maintain greenhouse gas balance and mitigate climate change (Bai et al., 2019; Tautges et al., 2019). Compared to natural ecosystems, soils in agroecosystems are generally depleted in SOC due to cultivation practices (Poeplau & Don, 2015; Poeplau et al., 2011). However, the challenge of enhancing SOC sequestration in agroecosystems can be potentially achieved through effective management practices. Integrating cover crops (CCs) into agricultural rotations is one such strategy that can increase and maintain SOC in agroecosystems. Past research on CC‐mediated SOC changes has documented an increase in SOC from CCs (McClelland et al., 2021; Poeplau & Don, 2015), but to our knowledge, few studies have focused on understanding the SOC fractions where this new C is stored. A comprehensive understanding of the channeling of new C to different soil fractions is important for predicting the persistence and function of the sequestered SOC. The CC‐induced increases in SOC could arise from a variety of storage pathways, ranging from selective preservation of either plant or microbial necromass to increased occlusion of SOC in aggregates or mineral associations. Furthermore, the pathways of C accumulation could vary among the monocultures and mixtures of different CC functional types (legume, grass, brassica). A greater understanding of the controls on C accrual from CCs is critical for predicting the persistence of CC‐mediated SOC storage and designing agroecosystems for sustainable productivity and climate change mitigation.

Based on a meta‐analysis of 131 studies across the globe, including CCs into rotations led to an increase of SOC by 15.5% (Jian et al., 2020). However, despite exhibiting a promising potential to increase SOC, the CC‐induced effects on soil C sequestration and persistence are highly dependent on plant functional types (PFTs; grass, legume, brassica) and the associated quantity and quality of CC residues (Chahal et al., 2020; Jian et al., 2020). This is because the tissue quality of CC residues is a major factor regulating the microbial carbon use efficiency (CUE), which is the proportion of C substrate assimilated by the microbes relative to the C substrate respired as CO_2_ (Cotrufo et al., 2013). The CCs with high tissue quality (low C:N, e.g., legumes—C:N ratio, 8–20) would, in theory, have a higher microbial CUE compared with substrates of low tissue quality (high C:N; e.g., grasses—C:N ratio >25; Kallenbach et al., 2019). Thus, the CCs with high C:N ratio would result in a greater ratio of plant: microbial‐derived products in soil due to their lower microbial CUE. Although CCs are mostly grown in monocultures for their specific functions (legumes—fix atmospheric nitrogen [N]; grasses—scavenge nutrients; brassicas—suppress pests and pathogens), mixtures of CCs that belong to different PFTs are also widely promoted as they concurrently provide multiple ecosystem services (Finney & Kaye, 2017; Magdoff & Van Es, 2000; Reeves, 2018). However, compared to CC monocultures, the diversity of inputs from different PFTs in CC mixtures might give rise to variable microbial CUE. This in turn would result in the differential accumulation of SOC in different soil fractions, which is a lesser‐known aspect of CC mixtures.

Traditionally, much emphasis is placed on those CCs that increase the quantity of SOC, and higher plant biomass production is conventionally equated to greater soil C sequestration. For example, grass CCs are generally more effective at increasing the quantity of SOC than legume CCs due to higher biomass production and lower decomposition of grass CC residues (Blanco‐Canqui et al., 2015; Mazzoncini et al., 2011). However, a higher proportion of the SOC from grasses may accumulate in the light fraction or free particulate organic matter (fPOM) that is mostly composed of structural compounds not protected by mineral association or occlusion in aggregates (Cheng et al., 2011). This plant‐derived C can be easily decomposed under warming due to its high temperature sensitivity (Feng et al., 2008; Pisani et al., 2015) relative to C accrued in mineral fractions. In contrast, according to the microbial efficiency‐matrix stabilization (MEMS) hypothesis (Cotrufo et al., 2013), high‐quality litter (low C:N) would result in higher microbial products that can be stabilized in the mineral‐associated organic matter (MAOM) fraction. Thus, to mitigate climate change it is important to channel more SOC to protected soil fractions (Rocci et al., 2021). Although the main functions of CCs of different PFTs are well known and their differential ability to enhance soil C accrual has been measured in some cases, the effect of CCs in monocultures and mixtures on the accrual of SOC in different soil fractions and thus the persistence of SOC is remarkably less known. This knowledge gap is a hindrance in formulating CC practices to manage SOC in agroecosystems to effectively deal with global challenges.

Soil organic matter (SOM) comprises a multitude of biomolecules, which can be protected against decomposition by occlusion within soil aggregates or association with soil minerals (Helfrich et al., 2006; Lehmann et al., 2008; Liang et al., 2019; von Lützow et al., 2006). Accordingly, SOC is composed of organic molecules with different physical and chemical properties, stabilities, and turnover times (Angst et al., 2017; Sokol & Bradford, 2019). The total SOC in bulk soil is not always a sensitive indicator to detect changes in SOC stocks or to elucidate the mechanisms of SOC sequestration across soil management regimes (Heckman et al., 2021; Shao et al., 2019; Six, Callewaert, et al., 2002). Lavallee et al. (2020) recently put forth an elegant framework that separates soil C into meaningful components such as the POM and MAOM fractions to better understand SOM formation, persistence, and function. For instance, the fPOM, primarily consisting of easily decomposable plant components such as carbohydrates and aliphatic compounds, is characterized by rapid turnover times and lower stability (Schnecker et al., 2016; Six et al., 1998), while oPOM (occluded POM) protects organic matter by aggregation resulting in spatial inaccessibility to microbes and enzymes (von Lützow et al., 2006). However, POM and MAOM are distinct components of SOM with regard to their formation, stability, and function (Cotrufo & Lavallee, 2022). It is proposed that POM is predominantly of plant origin, with a relatively wider C:N ratio and higher inherent biochemical recalcitrance than MAOM. However, the MAOM can be mainly of microbial origin (Cotrufo & Lavallee, 2022) or both plant and microbial‐derived compounds can have equal contribution to MAOM (Angst et al., 2021) and can be stabilized through chemical bonding to minerals (Haddix et al., 2020). Therefore, distinguishing microbial versus plant contribution of POM and MAOM is instrumental to advancing our understanding and prediction of changes in SOC sequestration and stabilization under different cover cropping practices.

To investigate the effects of CCs belonging to different PFTs (legume, grass, and brassica) on the accrual and chemical composition of distinct SOM fractions, we took advantage of a long‐term organic grain crop‐CC rotation experiment in central Pennsylvania where 12 CC treatments consisting of legume, grass, and brassica in monocultures and mixtures were established in 2012. We determined the SOC content (mg C g^−1^ dry soil) of the bulk soil and of the three individual fractions, fPOM, oPOM, and MAOM. More importantly, we quantified the major plant‐derived (lignin, cutin, suberin, and long‐chain fatty acids) and microbial‐derived biomarkers (amino sugars and short‐chain fatty acids) in different SOM fractions to assess the input and sequestration of source‐specific SOM chemical composition. Based on the different properties of the three fractions and distinct traits among the three CC functional types, we hypothesized that (i) the cultivation of CC species characterized by low litter quality (i.e., high C:N ratio, e.g., grass) would have a greater proportion of C in the POM fraction, whereas the selection of CC species with high litter quality (i.e., low C:N ratio, e.g., legume) would have a greater proportion of C in the MAOM fraction. Further, from the perspective of SOC composition, we hypothesized that (ii) the cultivation of gramineous CCs would increase the plant‐derived compounds in POM compared with MAOM, whereas the cultivation of leguminous CCs would increase microbial‐derived compounds proportionately higher in MAOM than in POM. Considering the potential complementary characteristics contributed by CCs with different functional traits (Drost et al., 2020), we also hypothesized that (iii) the soils planted with CC mixtures with all the three functional types would be characterized with an intermediate proportion of plant‐ and microbial‐derived C components in POM and MAOM, respectively, compared to the soils under the respective monoculture CCs.

## 2 MATERIALS AND METHODS

### 2.1 Study site

The soils for this study were collected from an organic grain crop– (maize–soybean–winter wheat) CC rotation field experiment, at the Pennsylvania State University Russell E. Larson Agricultural Research Center, Rock Springs, PA (40° 43′N, 77°56′W, and 350 m a.s.l.) that has been maintained since 2012. The site has an average annual precipitation of 975 mm and mean monthly temperatures from −3°C in January to 22°C in July. The average annual minimum temperature is approximately −20°C, corresponding to the USDA plant hardiness zone of 6b. More than 80% of the experimental area is characterized as a Murrill channery silt loam (fine‐loamy, mixed, semiactive, mesic Typic Hapludult). The two other less dominant soil series present at the site are Hagerstown silt loam (fine, mixed, semiactive, mesic Typic Hapludalf) and Buchanan channery loam (fine‐loamy, mixed, semiactive, mesic Aquic Fragiudult; Murrell et al., 2017). Hapludalf and Hapludult were formed on late Pleistocene or similar aged deposits or areas of acid rock (Hapludult), while the Fragiudult was formed in loamy alluvium or residuum (Soil Survey Staff, 2017). All these soil types are classified as Luvisols according to the World Reference Base for Soil Resource (WRB; Schad & Dondeyne, 2016), and were deciduous broadleaf or mixed‐conifer‐broadleaf forests prior to historic cultivation. The surface soil (0–20 cm) texture is predominantly loam to clay loam (sand: 21%–40%; silt: 39%–48%; clay: 23%–33%; Amsili & Kaye, 2021; Kaye et al., 2019). The average soil pH and cation exchange capacity were 6.6 and 8.32 meq/100 g, respectively.

### 2.2 Experimental design

The experiment was conducted in a randomized, full‐entry complete block design with four replicates per treatment (Figure S1). Cash crops were planted in 3‐year maize–soybean–winter wheat rotation in main plots (24 × 348 m). Maize, soybean, and winter wheat were planted at 71,000 seeds ha^−1^, 450,000 seeds ha^−1^, and 5 million seeds ha^−1^, respectively. In July 2012, organic management was adopted, and the site was certified organic in 2016. Detailed management practices (e.g., fertilizer application) on cash crops were reported in the previous study from the same experimental site (Kaye et al., 2019). Briefly, dairy bed pack manure was applied at a rate designed to meet the phosphorus (P) requirements of the rotation. During the experimental period (2012–2019), manure was applied twice before planting winter wheat in 2015 and 2018, and prior to planting maize in the spring of 2013 and 2016. Total manure inputs across the 7 years were 750 kg N ha^−1^ and 370 kg P_2_O_5_ ha^−1^. Surface soil inorganic N concentrations in different CC treatments are provided in Table S1. Details of other management practices are provided in Supporting Information. The main plot was divided into 12 subplots (24 m × 29 m) where the 12 CC treatments were established in a randomized order. The 12 CC treatments included six CC monocultures, five CC mixtures, and a fallow (non‐CC control) treatment (Kaye et al., 2019). Due to the detailed characterization of the chemical composition of different soil fractions, this paper focuses only on three CC monocultures, a five species (5spp.) mixture, and the fallow. The three monocultures were selected from contrasting functional types: a legume: crimson clover (*Trifolium incarnatum* L., *Fabaceae*); a brassica: canola (*Brassica napus* L. cv. Wichita, *Brassicaceae*); and a grass: triticale (*X Triticosecale* Wittmack, *Poaceae*). The 5spp. mixture combined each of the above three component monocultures but with the addition of red clover (*Trifolium pratense* L.) and Austrian winter pea (*Pisum sativum*). However, red clover and Austrian winter pea were extremely rare in the 5spp. plots. Therefore, the 5spp. mixture was essentially a 3spp. mixture of canola, crimson clover, and triticale (Amsili & Kaye, 2021). The shoot and root C:N ratio of the CC treatments are provided in Table 1 and the proportion of biomass of different CCs in the mixture is provided in Table S2. Since 2012, CCs were planted during the third week of August after harvesting winter wheat and baling the straw. Before planting, a chisel plow with twisted shanks was used to incorporate wheat stubbles into the soil. The seedbed was then prepared by disk, s‐tine, and cultimulch operations (Amsili & Kaye, 2021). The CCs were seeded in rows spaced 19 cm apart with a no‐till drill fitted with a cone and belt seed distributor (Murrell et al., 2017). The fallow plots were managed as a weed‐free plots by surface tilling as needed in fall and spring. CC seeding rates were based on regional recommendations (Table S3).

**TABLE 1:** Average C:N ratios (±1 *SE*) of root and shoot tissues in different cover crop (CC) treatments over the 7‐year organic grain crop‐CC rotation

Functional type	CC treatment	Shoot C:N			Composite root C:N^a^		
		Fall	Spring	Average	Fall	Spring	Average
Grass	Triticale	18.3 (0.79)	25.9 (2.03)	22.1 (1.41)	35.5 (0.78)	40.6 (0.73)	38.1 (0.76)
Brassica	Canola	17.9 (0.86)	24.3 (1.23)	21.1 (1.04)	23.7 (0.62)	36.8 (0.79)	30.2 (0.71)
Legume	Crimson clover	12.7 (0.47)	12.8 (0.32)	12.8 (0.40)	16.9 (0.64)	15.9 (0.40)	16.4 (0.56)
Mixture	5Spp.Mix	13.8 (0.92)	15.4 (0.48)	14.6 (0.70)	21.0 (1.40)	30.8 (1.38)	25.9 (1.39)

^a^ The composite root C:N ratios were only measured in fall 2016 and spring 2017.

### 2.3 Biomass collection

The CC above‐and below‐ground biomass was sampled as per Murrell et al. (2017) and Amsili and Kaye (2021). Briefly, we sampled aboveground biomass in each fall, before species were winter‐killed but after the maximum fall growth, and in the spring before the termination of CCs. In each plot, the aboveground biomass was collected from three 0.25 m^2^ quadrats by clipping at the soil level and sorting by CC species. Amsili and Kaye (2021) calculated the root to shoot ratios in Fall 2016 and Spring 2017. Root biomass was sampled in aboveground biomass sampling quadrats by collecting a 10.3 cm diameter soil core to a depth of 40 cm using a Giddings tractor‐mounted‐hydraulic‐soil‐sampler from an in‐row and a between‐row location. Two in‐row cores and two between‐row cores were collected in each CC treatment subplot. Soil cores were then washed through a nested 2 and 0.5 mm sieve set and roots were picked from the POM above the sieves (Amsili & Kaye, 2021). Aboveground and belowground biomass samples were dried at 60°C for 1 week, weighed and analyzed for C and N concentrations by the combustion method as described in Finney et al. (2016).

### 2.4 Cumulative carbon input calculations

Cumulative C inputs for the entire rotation were estimated as per Amsili and Kaye (2021). Briefly, we calculated the total cumulative C inputs as the sum of C inputs from CCs and non‐CCs (including manure and unharvested cash crop biomass) during the experimental period (2012–2019). Before sowing winter wheat and maize, manure inputs were each ~3.8 Mg C ha^−1^. Cash crop C inputs were calculated based on harvest indices for wheat (0.42), maize (0.40), and soybean (0.55; Bolinder et al., 2007; Kemanian et al., 2007). We also incorporated approximate below‐ground contributions for all cash crops based on an assumed root to shoot ratio of 0.33 (Amsili & Kaye, 2021; Bolinder et al., 2007). The total C inputs from CCs were calculated by summing root and shoot C. The root C inputs of CCs were calculated based on the root‐to‐shoot ratios measured in root quadrats.

### 2.5 Soil sample collection

Soils were sampled in May 2019 before terminating the third entry of CCs. After removing the surface litter layer, soil samples from each of the five CC treatments were collected to a depth of 20 cm using a 2‐cm diameter soil corer. In each plot, six soil cores were collected and thoroughly mixed to obtain one composite sample that was immediately kept on ice and transported to the laboratory. Any non‐soil material and rock were manually picked out of the soil, and the soils were passed through a 2‐mm sieve. The soil samples were air‐dried and then oven‐dried at 50°C prior to further analyses.

### 2.6 Soil fractionation

To examine the effects of CC treatments on soil C pools and chemical composition, we separated the bulk soil into SOM fractions by size and density, which represent SOM pools with distinct mechanisms of formation, persistence, and functioning (Lavallee et al., 2020). The fractionation scheme was modified from the method described in Poeplau et al. (2018) and Mosier et al. (2019), resulting in three fractions: fPOM, sand‐sized oPOM, and silt clay‐sized MAOM. Briefly, a 10 g oven‐dried soil sample was density fractionated by suspending the soil with 35 ml of 1.85 g cm^−3^ sodium iodide (NaI) and centrifuging at 1250 *g* for 60 min. After centrifugation, the fPOM fraction (<1.85 g cm^−3^) was vacuum‐aspirated onto a 20‐μm nylon filter and rinsed multiple times with deionized water to remove any remaining NaI. The heavy fractions (>1.85 g cm^−3^) were then dispersed in a 0.5% sodium hexametaphosphate (NaHMP) solution by shaking for 18 h with glass beads on a reciprocating shaker at a low speed (200 oscillations per minute). This dispersion method can break macro‐ and micro‐aggregates (>53 μm; Six et al., 1998), confirmed by visual inspection during fractionation. Dispersed samples were passed through 53‐μm sieves and rinsed with deionized water to separate the oPOM (>53 μm) from the MAOM (<53 μm). It should be noted that the fraction >53 μm after density fractionation may include some heavy coarse organic matter according to the more recent definition of soil physical fractions (Samson et al., 2020). Nevertheless, for the simplicity of classification and considering its chemical composition, we still define the >53 μm fraction as oPOM in this study. All these three fractions were oven‐dried at 60°C till constant weight. Dried soil fractions were finely ground with a ball mill for elemental analysis. The SOC and total N contents in the bulk soil and all fractions were analyzed on an elemental analyzer (Carlo Erba NA 1500 Elemental analyzer: Thermo Scientific).

### 2.7 Characterization of chemical composition of SOC by biomarker analysis

Several analytical techniques, individually or in combination, are used to analyze SOC, each having pros and cons (Kögel‐Knabner, 2000). We used wet chemistry methods followed by chromatography‐mass spectrometry analysis to elucidate the source‐specific chemical composition of SOC. We measured the individual plant‐derived (lignin, cutin, suberin, and long‐chain fatty acids) and microbial‐derived biomarkers (amino sugars and short‐ chain fatty acids) in soil (Tamura & Suseela, 2021; Zhang & Suseela, 2021; Table 2).

**TABLE 2:** The soil biomarker compounds identified from biomarker analysis across all soil fractions of fPOM, oPOM, and MAOM

Origin	Classes	Compounds	Origin	Classes	Compounds
Plant derived	LFA	n‐Docosanoic acid	Plant‐derived	Suberin	α,ω‐Hexadecanedioic acid
		n‐Tetracosanoic acid			α,ω‐Octadecanedioic acid
		n‐Hexacosanoic acid			α,ω‐Docosanedioic acid
		n‐Docosanol			20‐Hydroxyicosanoic acid
		n‐Tricosanol			22‐Hydroxydocosanoic acid
		n‐Tetracosanol			24‐Hydroxytetracosanoic acid
		n‐Hexacosanol			26‐Hydroxyhexacosanoic acid
		Octacosanol		Cutin	Hydroxyhexadecanoic acid
		Triacontanol			9,10‐Dihydroxyoctadecanoic acid
		Nonacosane	Microbial‐derived	SFA	n‐Dodecanoic acid
		Hentriacontane			n‐Tetradecanoic acid
		Heptacosanoic acid			n‐Pentadecanoic acid
		Octacosanoic acid			n‐Hexadecenoic acid (16:1)
		Triacontanoic acid			n‐Heptadecanoic acid
	Lignin	p‐hydroxybenzaldehyde			n‐Octadecenoic acid (18:1)
		p‐hydroxyacetophenone		Amino sugar	Muramic acid
		Vanillin			Glucosamine
		Acetovanillone			Galactosamine
		p‐hydroxybenzoic acid	Others		n‐Eicosanoic acid
		Syringaldehyde			Pentadecanol
		Vanillic acid			Hexadecanol
		Acetosyringone			Octadecanol
		3,5‐dihydroxy‐benzoic acid			n‐Eicosanol
		Syringic acid			α,ω‐Nonanedioic acid
		p‐coumaric acid			Nonadecanoic acid
		Ferulic acid			Heneicosanoic acid

*Note*: Amino sugars were not extracted from the fPOM fraction due to the low mass recovery of fPOM.

Abbreviations: fPOM, free particulate organic matter; LFA, long‐chain fatty acids; MAOM, mineral‐associated organic matter; oPOM, occluded particulate organic matter; SFA, short‐chain fatty acids.

#### 2.7.1 Lipids and lignin monomers

Sequential chemical extractions such as solvent extraction, base hydrolysis, and copper (II) oxide (CuO) oxidation were conducted to isolate solvent‐extractable free lipids, hydrolyzable bound lipids, and lignin‐derived phenols, respectively (Otto & Simpson, 2007; Tamura et al., 2017; Zhang & Suseela, 2021). Briefly, air‐dried soil fractions (1 g oPOM or MAOM fraction; 10 mg fPOM) were sequentially extracted with 5 ml of methanol, dichloromethane: methanol (1:1; v/v), and dichloromethane in 15‐ml glass tubes. The three sequential extracts were combined into 50‐ml glass tubes, followed by the addition of 15 ml of deionized water to induce phase separation. The phase of dichloromethane located at the bottom layer was collected and stored at −20°C prior to analysis. The air‐dried soil residue in the initial 15‐ml glass tube was incubated at 95°C for 3 h after adding 5 ml of 1 N methanolic sodium hydroxide (NaOH) for the further extraction of hydrolyzable lipids. After cooling down, the supernatant was pipetted into a new 50‐ml glass tube, with the soil residue further extracted with 5 ml of dichloromethane: methanol (1,1; v/v). The two sequential extracts were combined with 62.5 μl of heneicosanoic methyl ester (C_21:0_; 100 μg/ml in methanol) that was spiked as an internal standard. The combined extracts were acidified to pH <2 by adding 1.5 ml of 6 m HCl. Liquid–liquid extraction was performed to retrieve hydrolyzable or bound lipids by adding 15 ml of deionized water. The dichloromethane phase at the bottom layer was collected and stored at −20°C prior to analysis.

After base hydrolysis extraction, CuO oxidation was performed on the air‐dried soil residue in the initial 15‐ml glass tube to isolate the lignin‐derived phenols. To mitigate oxidation losses of lignin phenol caused by low organic C content in some samples, all samples with <5 mg organic C was spiked with 10 mg of glucose as an additional C source for the following CuO oxidation (Kaiser & Benner, 2012). Briefly, the soil residue was transferred into a teflon‐lined acid digestion vessel and mixed with 1 g CuO, 150 mg ammonium iron (II) sulfate hexahydrate [Fe(NH_4_)_2_(SO_4_)_2_. 6H_2_O], and 15 ml of NaOH solution (2 m after flushing with argon). All vessels were sparged with pure argon for 5 min and then incubated at 155°C in an oven for 160 min. After cooling to room temperature, the products of lignin oxidation were spiked with 100 μl of the internal standard trans‐cinnamic acid (200 μg/ml) dissolved in methanol, and then acidified to pH <2 by adding 3 ml of 18 N H_2_SO_4_. After centrifugation for 10 min, 2 ml of ethyl acetate (pre‐cooled in ice) was added to induce phase separation. The phase of ethyl acetate at the upper layer was collected and stored at −20°C prior to analysis. Lignin‐derived phenols included cinnamyls (C; p‐coumaric acid and ferulic acid), syringyls (S; syringaldehyde, acetosyringone, and syringic acid), and vanillyls (V; vanillin, acetovanillone, and vanillic acid).

Aliquots of samples from the above sequential extractions were silylated with 100 μl of N‐methyl‐N‐(trimethylsilyl) trifluoroacetamide (MSTFA) combined with 1% trimethylchlorosilane (TMCS) at 60°C for 40 min. All silylated samples were analyzed on an Agilent 7980A gas chromatography (GC) system coupled to a 5975 C Series mass detector (Agilent Technologies). GC–MS operating conditions are specified in detail in Supporting Information. Individual compounds were identified based on authentic external standards, NIST library data, mass spectra, and fragmentation patterns reported in literature (Figure S2a–c; Table 2; Feng et al., 2010; Otto & Simpson, 2007; Tamura & Tharayil, 2014). External calibration curves graphed with authentic standards (14 phenolic compounds for lignin monomers, 1‐octadecanol for alkanols, 1‐tetradecanoic acid for alkanoic acid, and 16‐hydroxyhexadecanoic acid for hydroxy‐alkanoic acids) were used to quantify the concentration of identified compounds. The concentration of each compound was normalized to the SOC content in a specific soil fraction to reflect its relative proportion in SOC.

#### 2.7.2 Amino sugars

Considering the low mass recovery of the fPOM fraction and the assumed dominant contribution of plant‐derived C in fPOM (Cotrufo et al., 2019), which was also supported by the low concentration of short‐chain fatty acids in fPOM across all treatments in this study, amino sugars were only extracted and analyzed in oPOM and MAOM fractions. Three amino sugars (glucosamine [GluN], and galactosamine [GalN], and muramic acid [MurA]) were determined as per Appuhn et al. (2004) and Olofsson and Bylund (2016). Briefly, 0.5 g of air‐dried soil was hydrolyzed with 5 ml of 6 m HCl at 105°C for 6 h. After cooling to room temperature, the hydrolysate was filtered through a Whatman 0.45‐μm membrane filter (Whatman GF/A). A 0.4‐ml aliquot of the filtrate was evaporated at 40°C to remove HCl, re‐dissolved in 0.4 ml of deionized water, evaporated to dryness again, re‐dissolved in 1 ml of acetonitrile: ultrapure water (1:1), and then stored at −20°C prior to analysis.

We identified the three amino sugars based on accurate mass (<5 ppm error) and structural fragmentation of parent ions after collision‐induced dissociation (CID) with argon and using commercially available authentic standards (Muramic acid, D‐glucosamine hydrochloride, and D‐galactosamine hydrochloride; Figure S2d). After identification, we quantified these amino sugars on ultra‐fast liquid chromatography coupled to a triple‐quadrupole mass spectrometer (LCMS 8030; Shimadzu Scientific) with an ESI interface (Zhang & Suseela, 2021). Detailed LC‐ESI‐MS/MS conditions are provided in Supporting Information. Before quantification, we optimized the analyte m/z transition for individual amino sugars by performing multiple reaction monitoring (MRM). The final analyte m/z transitions for muramic acid, glucosamine, and galactosamine are [251.85 → 233.95, 216.00, 125.95], [179.90 → 161.95, 163.00, 72.05], and [179.90 → 162.10, 163.05, 72.15], respectively.

Microbial necromass C (the sum of bacterial necromass C and fungal necromass C) content was calculated by using the following equations (Joergensen, 2018; Liang et al., 2019):

(1) $$\text{Bacterial necromass}\;C\;\left(\mathrm{\mu g}\;g^{-1}\right)=\text{MurA}\times 45,$$

(2)

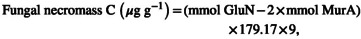

where 45 is the conversion value of MurA to bacterial necromass C; fungal necromass C was calculated by subtracting bacterial GluN from total GluN, assuming that GluN and MurA show a molar ratio of 2:1 in bacterial cells, where 179.17 is the molecular weight of GluN and 9 is the conversion value of fungal GluN to fungal necromass C (Engelking et al., 2007; Liang et al., 2019).

### 2.8 Statistical analysis

To compare the main and interactive effects of CC treatments (functional types) and soil fractions on the content and chemical components (lipids, lignin monomers, and amino sugars) of SOC, we applied a general linear mixed‐effects model using the “lme4” package, with the functional type and soil fraction considered as fixed effects and the block treated as a random effect. Considering that different soil fractions derived from the same initial soil sample are not independent, we also treated the functional type nested within the block (PFT*block) as a random effect. A linear mixed‐effects model was also used to compare the cumulative C inputs and SOC content in the bulk soil across CC treatments, with the functional type as a fixed effect and the block as a random effect. Statistically significant differences (*p* < .05) were further subjected to post hoc Tukey's HSD multiple comparison test using the “multcomp” package. A generalized linear model with normal distribution was applied to evaluate the relationship between the cumulative C inputs from CCs and the total cumulative C inputs. Similar linear regression was used to evaluate the relationships between the concentration of total microbial necromass C and the concentration of short‐chain fatty acids in specific soil fractions. For all statistical tests, we evaluated model assumptions of normality and homogenous variance using plots of residuals. When assumptions were not met, we applied log transformations to response variables or modeled heterogeneous variances using the “nlme” package (Pinheiro et al., 2017). Principal component analysis (PCA) was used to visualize the overall profiles and variances of SOC chemical composition across CC treatments in specific soil fractions using the “ade4” packages (Dray & Dufour, 2007). We also compared the CC treatment effects using a permutational multivariate ANOVA (PERMANOVA) with 999 permutations in the ‘vegan’ package (Anderson, 2001). To better visualize the differences in the specific SOC biomarkers across CC treatments in each soil fraction, the two‐way hierarchical clustering analysis (HCA) presented in heatmaps was performed using “pheatmap” package (Kolde & Kolde, 2018). HCA consists of calculating the dissimilarity, usually called the distance (Euclidean distance), between the individuals or samples, with one individual/sample corresponding to one column of the data matrix and with the composition variables (e.g., concentrations of metabolites, proteins, or biomarkers) corresponding to the rows of the data matrix (Meunier et al., 2007; Suseela et al., 2015). All statistical tests were performed using R version 4.0.2 (R Core Team, 2017).

## 3 RESULTS

### 3.1 Cumulative carbon inputs

**FIGURE 1:**
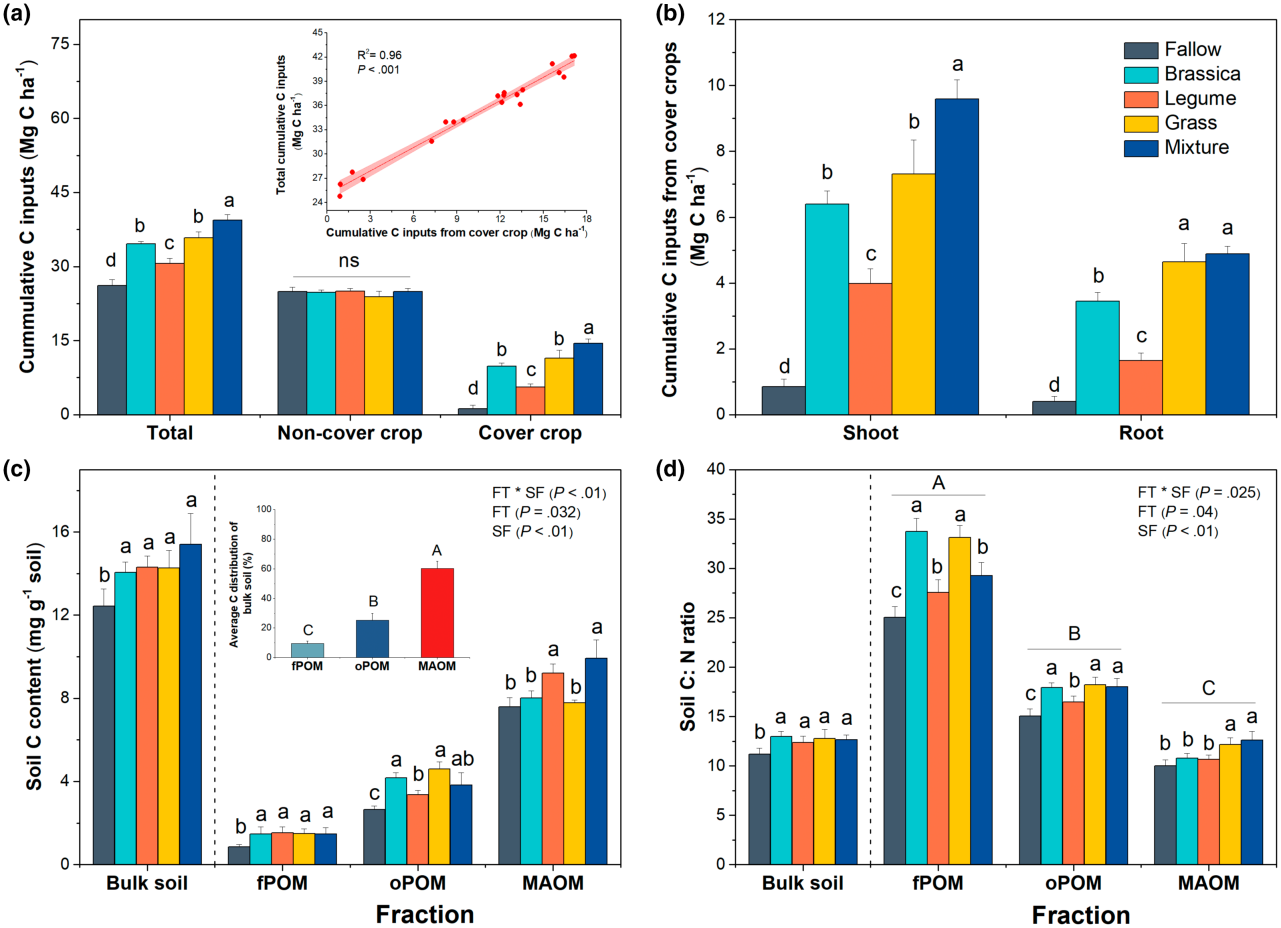
Cumulative C inputs (a, b), organic C content (c), and C:N ratio (d) of soils under different cover crop (CC) treatments during the 7‐year organic grain rotation from 2012 and 2019; (a) cumulative C inputs from non‐CCs and CCs; (b) shoot and root cumulative C inputs from CCs; (c) organic C content (mg g^−1^ soil) and (d) C:N ratio of bulk soil, free particulate organic matter (fPOM), occluded particulate organic matter (oPOM), and mineral‐associated organic matter (MAOM). The inserted plot in (a) shows the linear relationship between the CC cumulative C inputs and the total cumulative C inputs. The inserted plot in (c) shows the average C distribution of fPOM, oPOM, and MAOM in bulk soil. In the main plot of (c), error bars are ±1 *SE* of the mean (*n* = 4) with lowercase letters above bars indicating significant differences among CC treatments in bulk soil or specific fraction at *p* < .05. In the inserted plot of (c), error bars are ±1 *SE* of the mean (*n* = 20) with uppercase letters above bars indicating significant differences among soil fractions across all CC treatments at *p* < .05. In (a) and (b), error bars are ±1 *SE* of the mean (*n* = 4) with lowercase letters above bars indicating significant differences among CC treatments at *p* < .05. In (d), different uppercase letter above bars indicates significant differences among soil fractions across all CC treatments at *p* < .05. FT, functional type; FT × SF, interaction effect of functional type and soil fraction; ns, non‐significant; SF, soil fraction.

Over the 7‐year organic grain crop‐CC rotation, the total cumulative C inputs to soil ranged from 27.8 Mg C ha^−1^ (fallow) to 39.4 Mg C ha^−1^ (mixture; Figure 1a). The total C inputs were highest in the mixture followed by monocultures of brassica and grass, which had greater C inputs than the legume monoculture and the fallow treatment had the least C inputs (*p* < .05; Figure 1a,b). The cumulative C inputs from non‐CCs (including cash crops and manure) did not vary across the treatments (Figure 1a). The cumulative C inputs from CCs ranged from 1.2 Mg C ha^−1^ (fallow) to 14.4 Mg C ha^−1^ (mixture), following a trend similar to the total C inputs (Figure 1a) and correlating strongly with the variation in total C inputs among CC treatments (*R* ^2^ = .96, *p* < .001; Figure 1a). Within the cumulative C inputs from CCs, shoot C and root C had an average contribution of 65.6% and 34.4%, respectively, and followed a pattern similar to the cumulative CC C inputs across CC treatments (Figure 1b).

### 3.2 Soil fraction yield and composition

The soil mass recovery and total C recovery after fractionation were 97.6 ± 1.5% and 95.8 ± 3.5%, respectively, across all CC treatments. The fPOM, oPOM, and MAOM fractions contributed to 0.32%, 30.93%, and 66.35% of the bulk soil mass, respectively, across all treatments (Figure S3). Compared to the fallow, there was a higher fraction mass in fPOM in all CC treatments (*p* < .05). No significant difference of fraction mass was observed in oPOM or MAOM among all treatments (Figure S3).

### 3.3 SOC content and C:N ratio in bulk soil and soil fractions

Across all treatments, MAOM had the highest contribution (60.3%) to the SOC of bulk soil, followed with oPOM (25.2%) and fPOM (9.6%) (*p* < .01; Figure 1c). The SOC content in bulk soil and fPOM were, on average, 16.7% and 73.6% higher in all four CC treatments, respectively (*p* < .05) compared to the fallow while, no significant variation of SOC content was detected among the four different CC treatments (*p* > .05; Figure 1c). There was an evident effect of CC PFT on SOC content in both oPOM and MAOM (*p* < .05). The SOC content in oPOM were, on average, 21.2% and 33.6% higher in the brassica monoculture and grass monoculture, respectively, relative to the legume monoculture (*p* < .05). The mixture had similar SOC content in oPOM compared to monocultures of grass, brassica and legume. In contrast, the SOC content in MAOM were 16.8% and 25.9% higher in the legume monoculture and the mixture, respectively, compared to the monocultures of brassica and grass (*p* < .05; Figure 1c).

The C:N ratio in different soil fractions varied in the order: fPOM (25–34) > oPOM (15–18) > MAOM (10–13; *p* < .01; Figure 1d). Similar to SOC contents, the C:N ratios in bulk soil were, on an average, 13.3% higher in all CC treatments compared to the fallow (*p* < .05), with no significant variation of SOC content observed among the four CC treatments (*p* > .05; Figure 1d). The effects of PFT on C:N ratio were significant in all fractions. Specifically, in both fPOM and oPOM, the C:N ratios were higher in the monocultures of brassica and grass, as compared to the legume monoculture (*p* < .05). In contrast, compared to the monocultures of legume and brassica, the grass monoculture and the mixture had a mean of 15.6% higher C:N ratio in MAOM (*p* < .05; Figure 1d).

### 3.4 Plant‐ and microbial‐derived biomarkers in soil fractions

**FIGURE 2:**
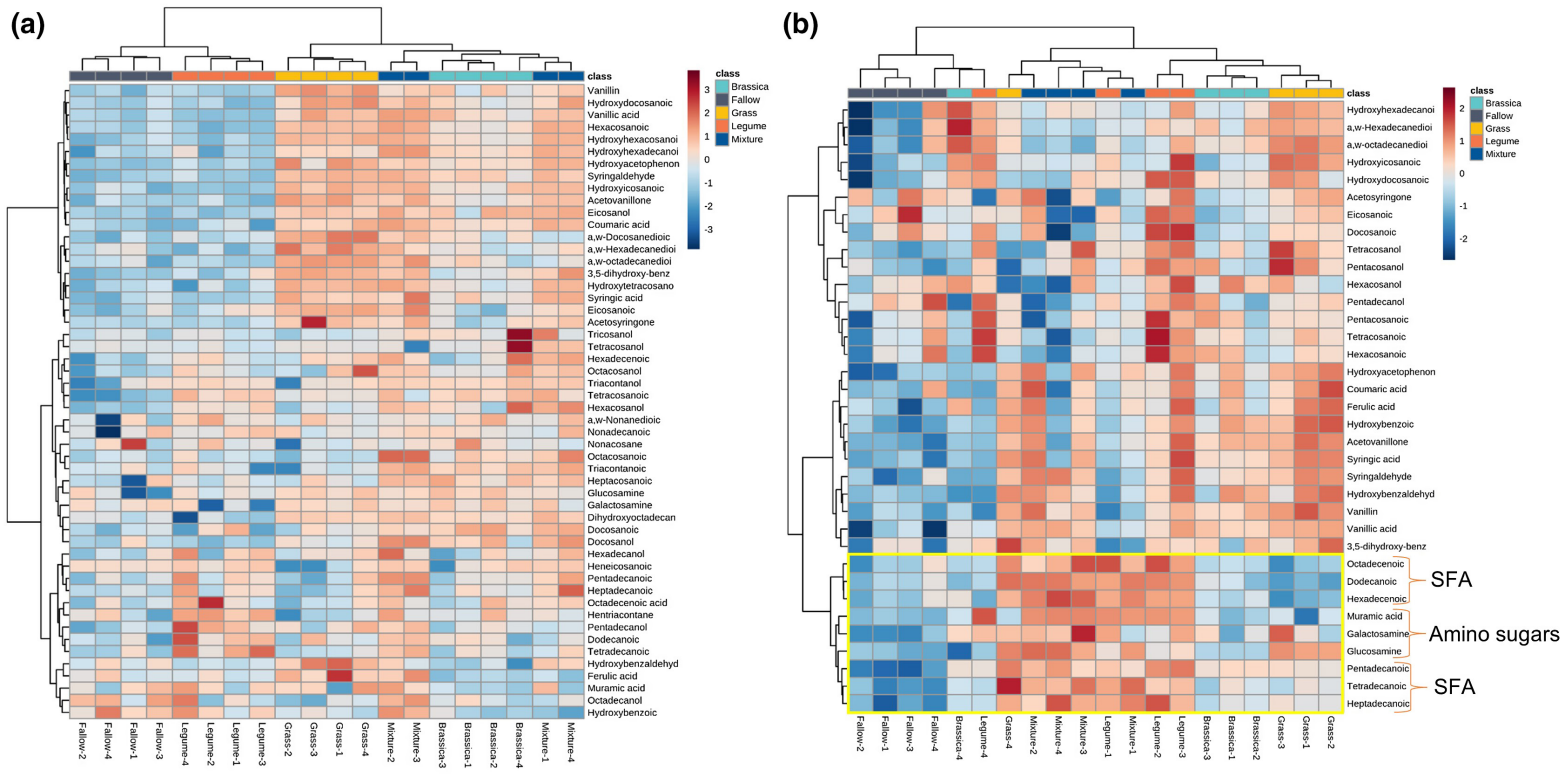
Heatmap and two‐way hierarchical clustering of the concentrations of all identified soil biomarkers in (a) occluded particulate organic matter (oPOM) and (b) mineral‐associated organic matter (MAOM) of soils under different cover crop (CC) treatments. Each column represents a sample from a specific CC treatment, and each row represents a positively identified soil biomarker based on mass spectrum information. The red and blue colors in different cells indicate relatively higher and lower concentrations of specific biomarkers in each sample, respectively. In (b), biomarkers belonging to short‐chain fatty acids (SFA) and amino sugars are highlighted, showing a dominant separation of soil organic carbon chemical composition in MAOM between CC treatments based on microbial‐derived biomarkers.

Cover crop treatments significantly influenced the profile of SOC chemical composition in different soil fractions according to PCA and PERMANOVA analysis (Figure S4). The hierarchical clustering of the concentrations of specific soil biomarkers further revealed different grouping patterns across CC treatments based on different compound classes (i.e., long‐chain fatty acids [LFA], cutin, suberin, lignin monomers, short‐chain‐fatty acids [SFA], and amino sugars; Figure 2; Figure S5; Table 2) in different soil fractions. Specifically, in oPOM, the legume monoculture and the fallow (which was devoid of much of the plant biomarkers) showed a clear separation from the mixture and the monocultures of grass and brassica, which had higher concentrations of plant‐derived biomarkers, especially suberin and lignin monomers (Figure 2a). In contrast, in MAOM, the legume monoculture and the mixture with higher concentrations of SFA and amino sugars clustered separately from grass and brassica (Figure 2b).

#### 3.4.1 Extractable plant‐derived biomarkers

**FIGURE 3:**
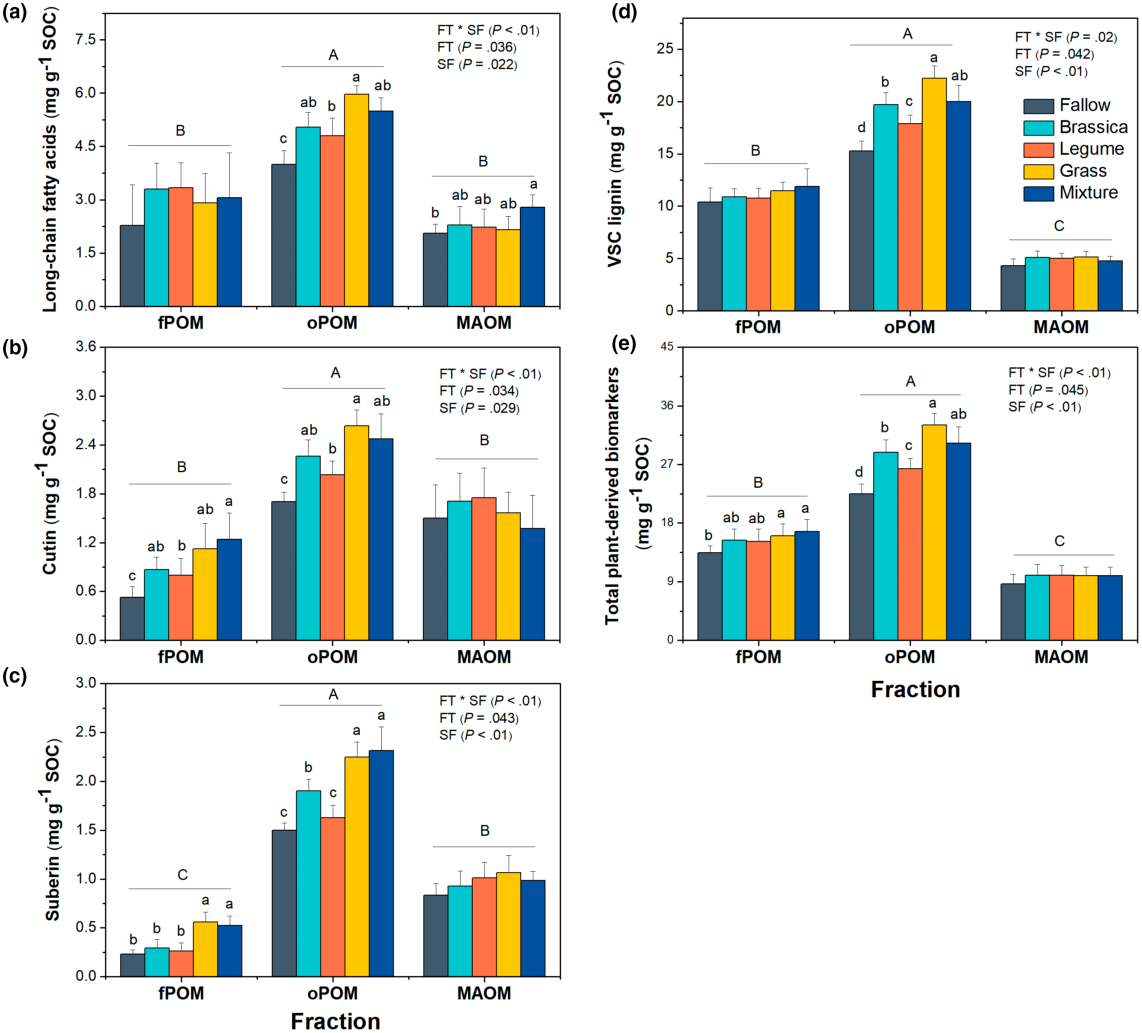
The concentrations of plant biomarkers (per gram organic carbon in soil fractions) in fPOM, oPOM, and MAOM; (a) long‐chain fatty acids (>C_24_ alkanes, >C_22_ n‐alkanoic acids and alkanols); (b) Cutin, (C_14_–C_18_ hydroxyalkanoic acids, C_16_‐di‐hydroxyalkanoic acids, *ω*‐hydroxy‐ and *ω*‐hydroxy‐epoxy alkanoic acids [C_16_–C_18_]); (c) suberin, (*α*,*ω*‐dicarboxylic acids (C_16_–C_24_; saturated and substituted) and *ω*‐hydroxyalkanoic acids [C_20_–C_30_; saturated and substituted]); (d) VSC lignin representing the sum of syringyl, vanillyl, and cinnamyl monomers of lignin and (e) total plant‐derived biomarkers (sum of LFA, cutin, suberin, lignin). Error bars are ±1 *SE* of the mean (*n* = 4) with lowercase letters above bars indicating significant differences between CC treatments in specific fraction at *p* < .05. Different uppercase letter above bars indicates significant differences among soil fractions across all CC treatments at *p* < .05. CC, cover crop; fPOM, free particulate organic matter; FT, functional type; FT × SF, interaction effect of functional type and soil fraction; MAOM, mineral‐associated organic matter; oPOM, occluded particulate organic matter; SOC, soil organic carbon; SF, soil fraction.

The concentrations of extractable plant‐derived biomarkers (Table 2) were higher in oPOM than in fPOM and MAOM across all CC treatments (*p* < .05; Figure 3). Compared to the legume monoculture and the fallow, the soils under the grass monoculture had higher LFA concentrations in oPOM (*p* < .05), whereas the LFA concentrations in fPOM and MAOM were similar across CC treatments (*p* > .05; Figure 3a). Cutin, primarily derived from leaf tissues, followed a trend similar to that of LFA, in which the soils under the legume monoculture had 29.5% lower cutin concentration than that under the grass monoculture in oPOM (*p* < .05; Figure 3b). The concentrations of root‐derived suberin in fPOM were higher in soils under the grass monoculture and the mixture than the respective fractions of the soils under other CC treatments (*p* < .05; Figure 3c). In oPOM, the grass monoculture and the mixture had a higher concentration of suberin followed by brassica whereas the fallow and legume monoculture had the lowest suberin concentration (*p* < .05; Figure 3c). The suberin concentrations in MAOM were similar across all CC treatments (*p* > .05; Figure 3c). Similarly, the concentration of VSC lignin phenols (the sum of V‐vanillyl, S‐syringyl, and C‐cinnamyl phenols) in oPOM was 23.8% higher in soils under the grass monoculture than that under legume, brassica and fallow (*p* < .05), while mixture had similar concentration of VSC lignin compared to grass and brassica. No difference of VSC lignin concentration was detected among all treatments in fPOM or MAOM fractions (*p* > .05; Figure 3d). The total plant‐derived biomarkers (sum of LFA, cutin, suberin, and lignin) in the oPOM was higher in grass monoculture compared to monocultures of legume and brassica and the fallow. Mixture had similar total plant‐derived biomarkers as that of grass and brassica monocultures (Figure 3e).

#### 3.4.2 Microbial‐derived biomarkers

**FIGURE 4:**
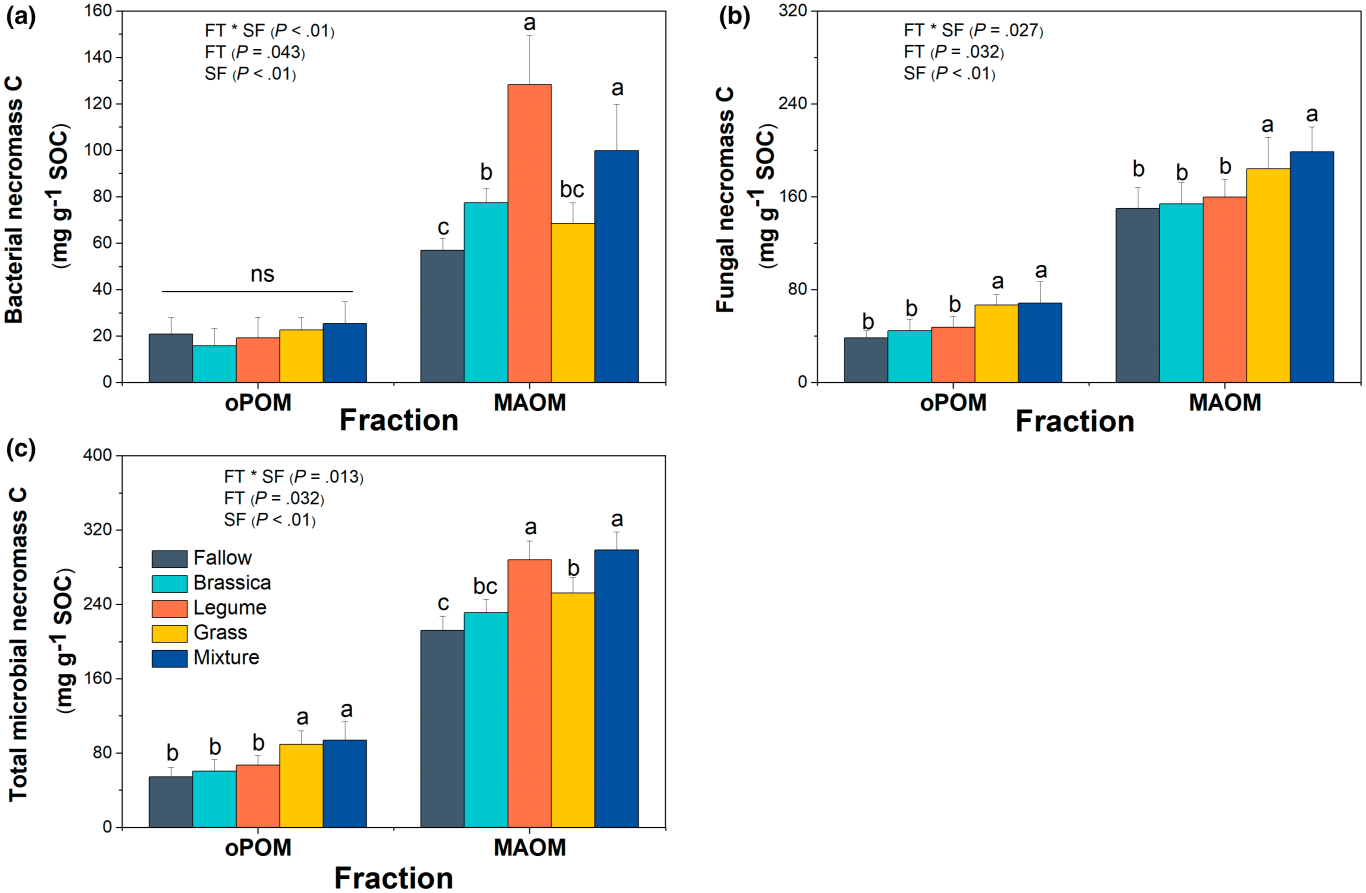
The concentrations of bacterial necromass C (a), fungal necromass C (b), and total microbial necromass C (c) (per gram organic carbon in soil fractions) in oPOM and MAOM. Error bars are ±1 *SE* of the mean (*n* = 4) with lowercase letters above bars indicating significant differences among CC treatments in specific fraction at *p* < .05. CC, cover crop; FT, functional type; FT × SF, interaction effect of functional type and soil fraction; MAOM, mineral‐associated organic matter; ns, non‐significant; oPOM, occluded particulate organic matter; SOC, soil organic carbon; SF, soil fraction.

**FIGURE 5:**
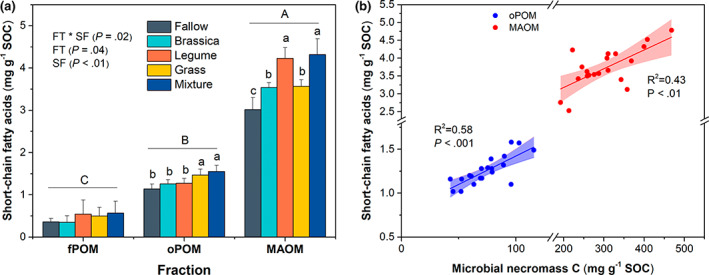
The concentration of short‐chain fatty acids (per gram organic carbon in soil fractions) in fPOM, oPOM, and MAOM (a) and the linear relationship between concentrations of microbial necromass C and short‐chain fatty acids across CC treatments (b). Short‐chain fatty acids, (C_10_–C_18_ *n*‐alkanoic and *n*‐alkaenoic acid). In (a), error bars are ±1 *SE* of the mean (*n* = 4) with lowercase letters above bars indicating significant differences among CC treatments in specific fraction at *p* < .05. Different uppercase letter above bars indicates significant differences among soil fractions across all CC treatments at *p* < .05. CC, cover crop; fPOM, free particulate organic matter; FT, functional type; FT × SF, interaction effect of functional type and soil fraction; MAOM, mineral‐associated organic matter; oPOM, occluded particulate organic matter; SOC, soil organic carbon; SF, soil fraction.

To trace the microbial origin of SOC sequestration, we calculated the bacterial and fungal necromass C using the biomarkers of amino sugars. In oPOM, there was no difference of bacterial necromass C concentration among all treatments (*p* > .05; Figure 4a). The concentration of bacterial necromass C in MAOM was similar between the soils under the legume monoculture and the mixture, which were on average 69.4% higher than that of other CC treatments (*p* < .05; Figure 4a). In contrast, compared to the monocultures of legume and brassica, the grass monoculture and the mixture had a mean of 22.8% higher concentration of fungal necromass C in MAOM (*p* < .05; Figure 4b). The concentration of fungal necromass C in oPOM showed a similar pattern to MAOM across all treatments (Figure 4b). Overall, the concentration of total microbial necromass C in MAOM was 21.2% higher in the soils under legume monoculture and the mixture relative to the soils under the monocultures of grass and brassica (*p* < .05; Figure 4c). In addition, the concentration of total microbial necromass C was 3–4 times higher in MAOM relative to oPOM across all CC treatments (*p* = .01; Figure 4c). The concentration of SFA (characteristic of microbial biomass) only varied among CC treatments in oPOM and MAOM rather than fPOM (Figure 5a) and followed a similar pattern as that of total microbial necromass C (Figure 4) and had a linear correlation with microbial necromass C (Figure 5b).

## 4 DISCUSSION

Cover crops have long been recognized to play an important role in conserving or increasing soil C sequestration by providing additional OC inputs to the soil (Sainju et al., 2007). However, the effects of different CCs belonging to different PFTs on the quantity and composition of physiochemically associated pools of SOC are remarkably less investigated. It is imperative to understand the formation, persistence, and function of CC‐mediated SOC in agroecosystems to build a SOC pool that is resilient to global changes as well as maintain the soil fertility to sustain agroecosystem productivity. Our results revealed divergent responses of the quantity and chemical composition of SOC in different soil fractions, providing evidence for shifting C dynamics following the inclusion of varied CC functional types. To our knowledge, our study for the first time revealed that the mixtures of CCs with all three functional types such as grass, brassica and legume enhanced SOC in both oPOM and MAOM fractions, where the oPOM was dominated by plant‐derived compounds while MAOM was dominated by microbial‐derived compounds. This microbial C can be stabilized by association with minerals increasing the long‐term C sequestration. (Lavallee et al., 2020). The mixtures of all three‐CC functional types can also increase the short‐lived soil C such as oPOM. Farmers could maintain these C stocks over time through repeated inclusion of CCs in rotation and thus would be an effective strategy to draw down C from the atmosphere. Additionally, these mixtures can increase the functioning (soil fertility) of SOC through enhanced C sequestration in the MAOM fraction, which can be a source of N to microbiota (Castellano et al., 2012) as some of the N from MAOM becomes available on short time scales (Jilling et al., 2018). Since the strategies for climate change mitigation through management of SOM should include both SOC sequestration and functioning (Lavallee et al., 2020), our study thus provides novel insights into both these aspects of SOC management in agroecosystems. Moreover, our results provide one of the first tests of a new fundamental biogeochemical theory (i.e., MEMS hypothesis; Cotrufo et al., 2013) in a real‐world setting describing how plant residues of different quality enrich distinct SOM fractions.

### 4.1 CC functional types differentially influence the accrual of SOC in POM and MAOM fractions

The CC‐induced positive effect on the SOC accumulation (Figure 1c) could be mainly attributed to the increased cumulative C inputs in all CC plots (Figure 1a). Similar results have been reported in many previous empirical and modeling studies (Büchi et al., 2018; Chahal et al., 2020; Schipanski et al., 2014; Tautges et al., 2019). Although manure application in organic systems can increase SOC, in our system, manure application did not mask the effect of CCs as all CC treatments including fallow had similar manure application. Our results indicated that the contribution of cumulative C inputs to SOC accrual was largely dominated by CC C inputs rather than non‐CC inputs (Figure 1a) over the study duration, which is consistent with the findings in previous studies (Chahal et al., 2020; Garcia‐Palacios et al., 2018). Since the pre‐treatment SOC contents (1.29%) were similar across all study plots, the observed greater SOC content with CCs than without, provides evidence for the enhanced C storage potential in agriculture systems with CCs.

The plant‐derived macromolecules have been demonstrated to have lower associations with the inner‐surface minerals compared to the microbial‐derived, less complex biomolecules (Lehmann & Kleber, 2015; Tamura et al., 2017). Therefore, we hypothesized that the inclusion of CCs with high‐quality litter (e.g., low C:N ratio) would result in more C accrual in MAOM than in POM. This hypothesis was supported by the greater C content in MAOM but lower C content in oPOM of soils under the legume (crimson clover) monoculture and the mixture relative to the monocultures of grass (triticale) and brassica (canola; Figure 1c). The higher C content in MAOM of soils under the legume monoculture, despite the lower cumulative C inputs, could potentially result from the higher rate of in vivo processing of labile litter inputs through the “microbial C pump” (Cotrufo et al., 2013; Liang et al., 2017), thus enhancing the ability of plant C inputs to associate with the soil mineral fraction (Grandy et al., 2008). In addition, due to the ability of legumes to fix atmospheric N, high‐N litter inputs may shift the stoichiometry of substrates closer to meeting the microbial N demand. (Lavallee et al., 2020). This could shift SOC formation from POM to MAOM and increase POM decomposition due to increasing N availability (Cotrufo et al., 2013; Lavallee et al., 2020). Previous studies have reported a similar increase in MAOM formation and POM decomposition due to N addition (Bradford et al., 2008; Kirkby et al., 2014).

Consistent with the MEMS hypothesis wherein lower quality litters should favor the formation of POM over MAOM (Cotrufo et al., 2013), our results indicated a greater C content in oPOM of soils under the monocultures of grass and brassica, which is also similar to the results observed in a litter manipulation study with low‐quality litter (Crow et al., 2009). However, although light fraction organic matter is considered to be most sensitive to changes in crop inputs (Ding et al., 2006; Six et al., 1999), we did not observe differences in C content in fPOM of soils under different CC functional types. Nevertheless, given that CC residues in fPOM present a short‐term SOC pool and contribute little to SOC stabilization (Austin et al., 2017; Dungait et al., 2012), patterns of C change in oPOM and MAOM may better reflect the long‐term SOC persistence influenced by crop inputs through physical protection within soil aggregates and mineral association. Overall, our results highlighted the contrasting effects on the changes of C content in oPOM and MAOM fractions induced by different CC functional types and supported the recently proposed framework claiming independent formation and persistence of POM and MAOM in soil (Cotrufo et al., 2015; Haddix et al., 2020).

### 4.2 Influence of CC functional types on the composition of soil particulate organic and mineral associated C

Plant litter inputs characterized by distinct quality (C:N) and chemical composition will have cascading effects on the quantity and composition of plant‐ and microbial‐derived components in soil (Jia et al., 2019). Since these plant‐ and microbial‐derived compounds differ in their propensity to associate with soil physical fractions, they may differentially influence the pathways of soil C formation and stabilization (Tamura et al., 2017). Consistent with our second hypothesis, the soils under the grass monoculture exhibited a higher proportion of plant‐derived biomarkers (plant lipids and lignin) in oPOM compared to the soils under the legume monoculture (Figure 3). The slower decomposition rate of complex structural plant compounds and selective preservation in the physically protected POM, coupled with the overall higher cumulative litter inputs in soils under the grass monoculture, would explain the apparent persistence of these compounds in oPOM (Helfrich et al., 2006; Tamura et al., 2017). In addition, the higher rooting density and fibrous root architecture of the gramineous CCs (Amsili & Kaye, 2021) would facilitate better soil aggregation, which could in turn provide greater protection of plant compounds in oPOM (Tamura et al., 2017). Since there was no significant difference in cumulative CC C inputs or shoot C:N ratio between the monocultures of grass (C:N‐22) and brassica (C:N‐21), the greater concentration of lignin in oPOM of the soils under the grass monoculture relative to the brassica monoculture (Figure 3d) would have potentially resulted from the higher root biomass and higher C:N ratio of triticale roots (38; root diameter: <2 mm) compared to canola roots (30; Figure 1b; Table 1; Amsili & Kaye, 2021). This argument is further supported by the greater concentration of root‐derived suberin in oPOM of the soils under the grass monoculture (Figure 3c).

Compared to POM, MAOM has been reported to have fewer plant‐derived compounds but more microbial‐derived products (Poirier et al., 2005), which is supported by the lower C:N ratio and higher (3–4 times) concentration of microbial necromass C in MAOM relative to oPOM across all CC treatments (Figures 1d and 4c). Consistent with our expectation, the soils under the legume monoculture exhibited a higher concentration of microbial necromass C in MAOM compared to the soils under monocultures of grass and brassica (Figure 4). Greater retention of microbial necromass C under legume cultivation may be caused by more active assimilation and transformation of labile plant‐derived C into microbial biomass through the “in vivo microbial turnover pathway”, leading to higher microbial necromass that can efficiently associate with MAOM (Lavallee et al., 2020; Liang et al., 2017). This interpretation could be corroborated by the positive correlation between the concentration of total microbial necromass C and microbial biomass (as indicated by SFA; Figure 5b). Legumes have larger positive effects on soil microorganisms than grasses and forbs (Spehn et al., 2000; Zhao et al., 2015), which could be primarily attributed to the higher quality of C inputs from litter and rhizodeposits of legumes (Fustec et al., 2010).

Despite the greater total microbial necromass C detected in MAOM of the soils under the legume monoculture, the patterns of soil bacterial and fungal necromass C differed between CC functional types (Figure 4a,b), which appeared to be associated with the changed soil microbial community structure. A previous CC study conducted at the same experimental site reported lower fungi to bacteria ratios in legume monocultures relative to grass monocultures and mixtures (Finney et al., 2017). The different responses of bacteria and fungi to legume and nonlegume CCs have been well documented (Frasier et al., 2016; Muhammad et al., 2021; Nakamoto et al., 2012). Bacteria favor the decomposition of high‐quality litter, such as those of legume CCs, while fungi are more efficient in utilizing relatively low‐quality less labile litter, such as from gramineous CCs (Viketoft et al., 2009; Wardle et al., 2006). The higher C:N ratio in MAOM fraction from grass monoculture and mixtures compared to legume and brassica mirror the proposed variation in soil microbial community structure (Figure 1d) since C:N ratio of fungi (4.5–15) is typically higher than bacteria (3–5; Paul, 2014; Cotrufo et al., 2019). Furthermore, the extra N inputs from legumes may have inhibited the growth of soil fungi (De Vries et al., 2006; Mbuthia et al., 2015), thus impeding the accumulation of fungal necromass C. Compared with the grass monoculture, although receiving the same amount of cumulative C inputs with similar litter quality, the soils under the brassica monoculture had a lower concentration of fungal necromass C in MAOM (Figure 4b). This could be potentially due to the intrinsic anti‐fungal and non‐mycorrhizal traits associated with brassicas (Cosme et al., 2018; Murrell et al., 2019). Decomposing brassica litter has been reported to dramatically decrease soil fungal biomass and diversity (Hollister et al., 2013) and they are often used as “biofumigants” to suppress fungal pathogens (Vukicevich et al., 2016).

Since different CC species varied in their productivity, litter quality, and contemporary influence on soil microbial community, we expected that CC mixtures would balance the contrasting effects on the chemistries in specific SOC fractions between the monocultures of legume and grass. This hypothesis was mostly supported by the higher concentration of plant‐derived compounds in oPOM of soils under the mixture relative to the legume monoculture, and simultaneously greater accumulation of microbial necromass C in MAOM of soils under the mixture than that under the grass and brassica monocultures (Figures 3 and 4). This observation could be associated with the potential complementary effect of CC mixtures in balancing the contrasting traits of individual CC species, i.e., lower biomass production of legumes and lower litter quality of grasses (Table 1; Table S2; Amsili & Kaye, 2021). In addition, compared to monocultures, CC mixtures with a broader nutrient and stoichiometric spectrum could increase the niche breadth of substrates to the soil system, subsequently increasing the metabolic potential and microbial biomass and diversity (Baumann et al., 2009; Drost et al., 2020; Nicolardot et al., 2007), which may favor the SOC persistence in MAOM.

### 4.3 Implications for long‐term soil C accrual and stabilization through CCs

There has been a significant interest in the adoption of CCs to increase SOC stocks due to agricultural intensification. The main focus of our study was to evaluate the effects of different functional types of CCs on the content and composition of distinct SOC fractions. Our study shows that the inclusion of CCs with distinct functional traits has differential influences on the abundance of plant‐derived C and microbial necromass C that are distributed in POM and MAOM fractions. We found that CCs characterized by low litter quality (e.g., grass) resulted in a higher accrual of POM which was abundant in plant‐derived C, while CCs with high litter quality (e.g., legume) contributed to a greater accumulation of microbial necromass C in MAOM. Thus, our results support the two‐pathway model of SOM formation (Cotrufo et al., 2015; Tamura et al., 2017). Our results also showed that CC mixtures would balance the contrasting effects on the chemistries in POM and MAOM imposed by individual CC species with distinct traits. These results indicate that different functional types of CCs may affect dominant SOC formation pathways (broadly POM vs. MAOM), subsequently impacting short‐ and long‐term soil C stabilization and stocks. Thus, although the cumulative C inputs (both above‐ and below‐ground) from CCs were different among CC functional types (Figure 1b), SOC content in bulk soil did not differ between them, which indicates that the total plant C inputs alone do not explain the variation of SOC accrual in the bulk soil as the pathways of SOC formation and persistence differed among the CCs.

Based on the recent conceptual frameworks arguing the dominant role of MAOM formation in long‐lived SOC stabilization (Cotrufo et al., 2013; Lavallee et al., 2020), targeting MAOM for SOC sequestration by selecting CCs characterized by functional traits with high‐quality litter residues that favor the formation of MAOM over POM may be more promising, from a persistence perspective. However, it is important to note that, although the sequestration of long‐lived soil C is primarily focused on, short‐lived soil C should not be neglected, which could be managed to effectively capture atmosphere C through rapid biomass production despite its short retention (Lavallee et al., 2020). From this perspective, our study highlights that CC mixtures might be a feasible strategy to target both POM and MAOM by complementing advantageous functional traits from different species. Nevertheless, the complementary effect of CC mixtures depends largely on the right selection and combination of CC species (Fox, 2005; Saleem et al., 2020), since the potential competitive or antagonistic interactions between species may hinder the expression of traits from specific functional types (Murrell et al., 2017). Therefore, future research on identifying the CC functional traits and their compatibility in mixtures is required to formulate knowledge‐based practices to enhance the C sequestration potential of CC mixtures. Although our study was conducted in an organic system, the cash crop rotation and CC species we used are also widely used in conventional agriculture. Furthermore, because the manure did not mask the effect of CCs, similar patterns in SOC quantity and composition due to CCs are likely to be observed in conventional farming systems with similar site characteristics and crop‐CC rotations. Future studies may explore how the addition of chemical fertilizers and differences in management practices such as tillage in conventional versus organic farming systems influence the effect of different CC PFTs on SOC dynamics in soil physical fractions at varying depths. Overall, our study highlights the importance of plant trait‐based understanding and predictions of cover cropping practices to improve SOC stocks and soil health, which can also provide guidance for land managers and policymakers.

## AUTHOR CONTRIBUTIONS

Vidya Suseela and Ziliang Zhang conceived the idea of the study. Jason P. Kaye designed and carried out the CC field experiment. Ziliang Zhang performed soil physical fractionations, soil biomarker analysis, and data analysis. Brosi A. Bradley and Joseph P. Amsili collected plant and soil samples and helped Ziliang Zhang in analyzing data on the cumulative C inputs. Ziliang Zhang wrote the first draft of the manuscript and all co‐authors contributed to the revisions.

## CONFLICT OF INTEREST

The authors have no competing interests that might be perceived to influence the results and discussion reported in this paper.

## Supporting information


Appendix S1.

## Data Availability

The authors confirm that all data related to this publication is available via the Dryad Digital Repository https://doi.org/10.5061/dryad.ncjsxksxj.
