# Supplementary material for: Cover crop functional types differentially alter the content and composition of soil organic carbon in particulate and mineral‐associated fractions
Source: Glob Chang Biol. 2022 Jul 18;28(19):5831–48. doi: 10.1111/gcb.16296 (PMC9545985; doi:10.1111/gcb.16296)
Supplement: Supplementary file 1 — Appendix S1. [file GCB-28-5831-s001.docx]

**Supplementary data for:**

**Cover crop functional types differentially alter the content of composition of soil organic carbon in particulate and mineral-associated fractions**

***Ziliang Zhang^1^*^†^*^*^, Jason P. Kaye^2^, Brosi A. Bradley^2^, Joseph P. Amsili^2ǂ^, Vidya Suseela^1*^***

^1^Department of Plant & Environmental Sciences, Clemson University, Clemson SC, USA

^2^Department of Ecosystem Science & Management, Pennsylvania State University, PA, USA

**^*^Correspondence**

[**vsuseel@clemson.edu**](mailto:vsuseel@clemson.edu)

[**ziliangz@illinois.edu**](mailto:ziliangz@illinois.edu)

**^†^**Present Address: Institute for Sustainability, Energy, and Environment, University of Illinois at Urbana-Champaign, Urbana, IL, USA.

***^ǂ^*** Present Address: Section of Soil and Crop Sciences, School of Integrative Plant Science, Cornell University, Ithaca, NY, USA.

**Number of text pages: 11**

**Number of figures: 6**

**Number of tables: 3**

**Notes S1 Additional materials and methods**

**Cover crop field management practices**

Management practices in the same study site were detailed in Kaye et al. (2019). Before planting maize, the manure and CC residues were incorporated using a moldboard plow and disc. Similarly, moldboard plow and disc were used to incorporate CC residues prior to planting soybean. The CC residues were flail mowed before ploughing to ensure effective incorporation. Before planting wheat, a chisel plow and disc were used to incorporate both manure and soybean residues. After these primary tillage operations, a seedbed for all crops was prepared with an s-tine field cultivator followed by a cultimulcher. Weed management was achieved in the maize and soybean with repeated passes of a tineweeder, rotary hoe, and inter-row cultivator. CCs were planted in August between wheat harvest and maize planting. Between maize harvest and soybean planting, the CCs were planted in late September or early October. The legume seeds were coated with N-Dure dry inoculant with the appropriate Rhizobia species before seeding. Cover crops were planted using an Almaco (Nevada, IA) Cone Plot Planter mounted on a double disc seed drill (Great Plains Manufacturing, Salina, KS) with 19 cm row spacing (Murrell *et al.,* 2017). Once per season, the fallow plots were surface tilled for weed management in fall and spring. The CCs were terminated using flail mowing and all CC treatments were terminated on the same day.

**GG-MS conditions for biomarker analysis**

The silylated samples were analyzed on an Agilent 7980A GC system coupled with a 5975 C Series mass detector. A DB-5 MS fused-silica capillary column (30 m length × 0.25 mm internal diameter × 0.25 μm film thickness) was used to separate the analytes. 1 μl of silylated sample was injected with a split (1:5 for both solvent extraction and base hydrolysis; 1:10 for CuO oxidation). The temperature was set as 60 ^o^C for 1 min followed by a linear ramp of 10 ^o^C per min to 300 ^o^C and held at 300 ^o^C for 10 min. The temperature for both the injection port and the mass spectrometer (MS) interface were kept at 270 ^o^C. The flow of carrier gas (He) was maintained at 1 ml min^-1^. The temperature of MS source and MS quad were maintained at 230 ^o^C and150 ^o^C , respectively. A constant gain of 1 (EMV = 1412V) was set for the electron multiplier. The scan speed was 2.97 scans sec^-1^ with the scan range of 60 to 550 amu.

**LC-ESI-MS/MS conditions for amino sugars**

A HILLIC-Z analytical column (Poroshell 120, 150 mm × 2.1 mm, 2.7 µm) was used to separate the amino sugars in a gradient elution mode using 20 mM ammonium formic acid (solvent-A) and 90% acetonitrile + 10% 20 mM ammonium formic acid (solvent-B). During the 8-minute analysis, concentration of solvent A was increased linearly from 10% to 40%. The solvent flow rate was maintained at 0.3 ml min^-1^. Nitrogen was used as the nebulizing gas (3 l min^-1^) and drying gas (10 l min^-1^) after heating to 250 ℃. The ionization voltage was 4500 V. 4 µl of sample was injected via the syringe that was washed between injections to exclude carry-over effects.

**References**

Murrell EG, Schipanski ME, Finney DM, Hunter MC, Burgess M, LaChance JC, et al. Achieving diverse cover crop mixtures: Effects of planting date and seeding rate. Agron J. 2017; 109(1):259–71.

**Fig. S1.** (A) Ariel photo of the experiemntal site at Rock Springs Agricultural Research Station, PA, USA. (B) Schematic map plot showing the specific cover crop treatments.


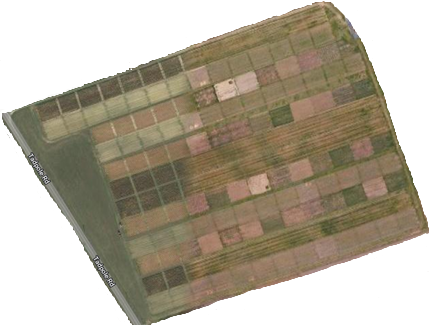


A

B


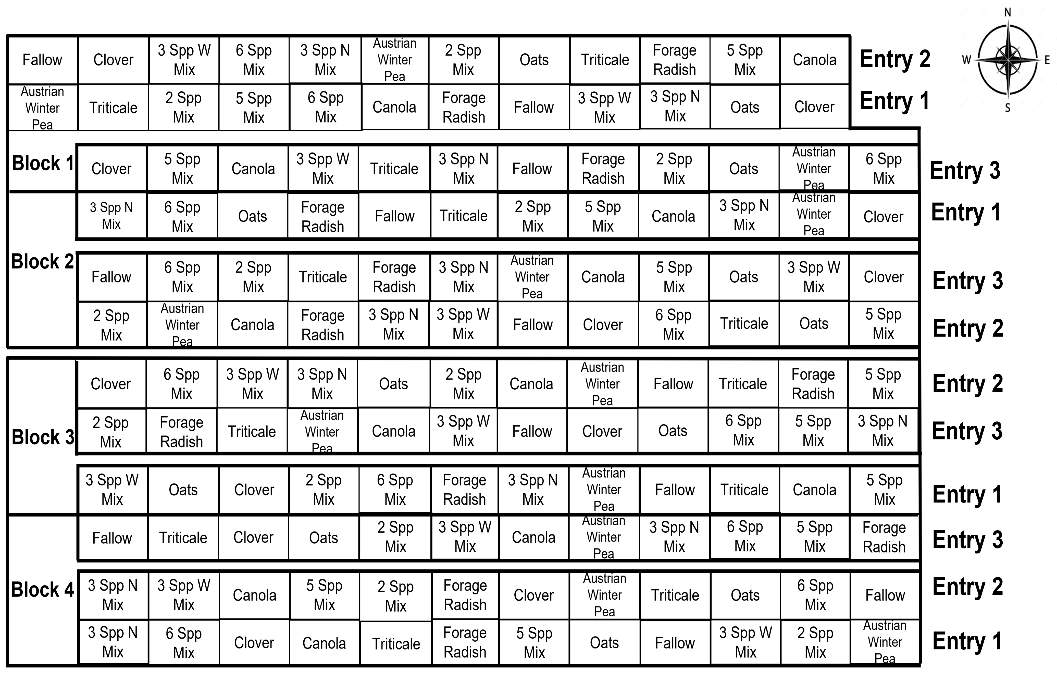


Block 1

Block 2

Block 3

Block 4


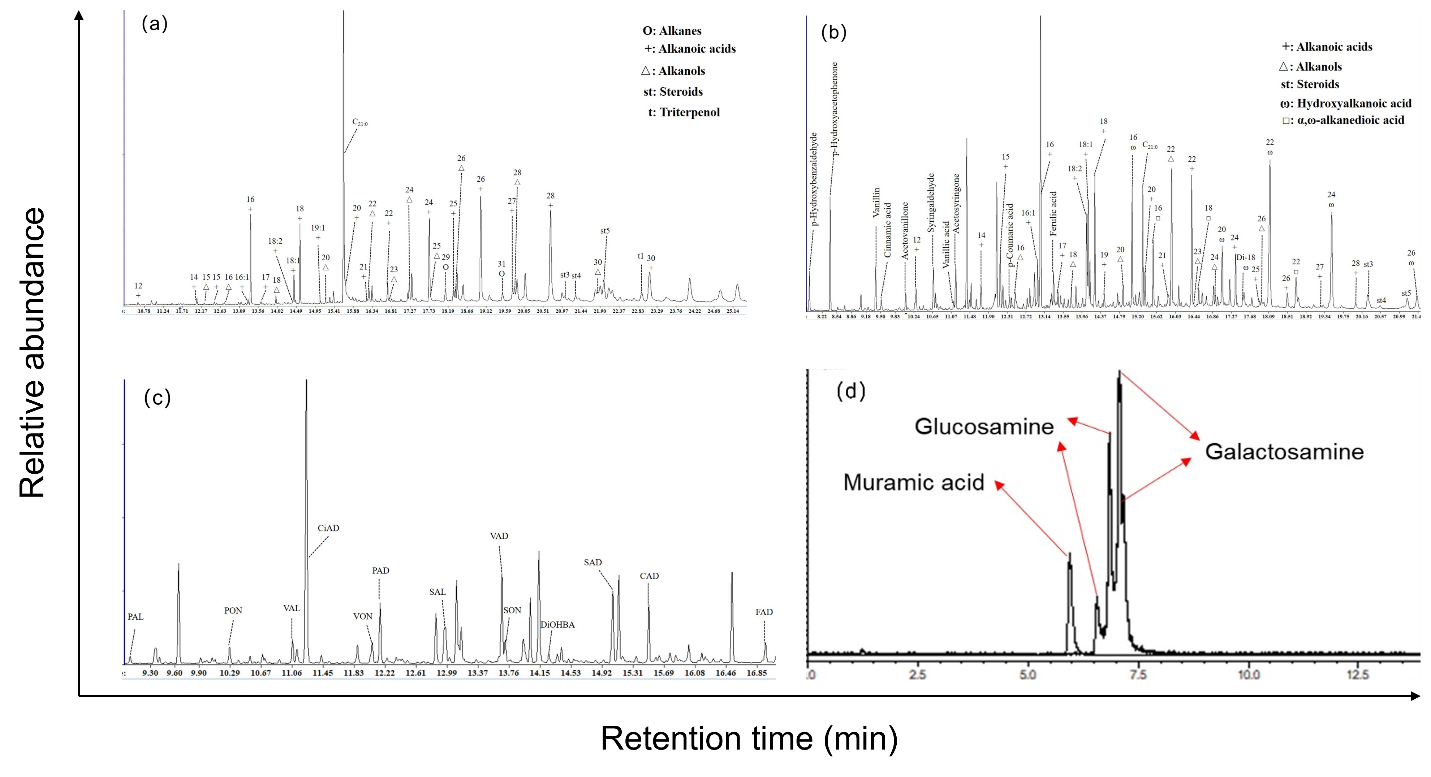


**Fig. S2.** GC-MS and LC-MS/MS chromatograms (TIC) of the plant- and microbial-derived biomarkers extracted from physical fractions of soils under different CC treatments. (a) Silylated solvent extracts; (b) Silylated base hydrolysis products; (c) Silylated CuO oxidation products; (d) Amino sugars (muramic acid, glucosamine, and galactosamine). In (a) and (b), + = n-alkanoic acids, △= n-alkanols, ○ = n-alkanes, st = steroids, 􀀀 = α,ω-alkanedioic acids, **ω =** Hydroxyalkanoic acids. Numbers refer to total carbon numbers in aliphatic lipid series. In (c), PAL, p-hydroxybenzaldehyde; PON, p-hydroxyacetophenone; PAD, p-hydroxybenzoic acid; DiOHBA, 3,5-dihydroxy-benzoic acid; VAL, vanillin; VON, acetovanillone; VAD, vanillic acid; SAL, syringaldehyde; SON, acetosyringone; SAD, syringic acid; CAD, p-coumaric acid; FAD, ferulic acid.

**Fig. S3.** Fraction mass of free particulate organic matter (fPOM) occluded particulate organic matter (oPOM), and mineral associated organic matter (MAOM) of soils under different CC treatments. Error bars are ±1SE of the mean (n = 4) with lowercase letters above bars indicating significant differences among CC treatments in specific fraction at *P* < 0.05. Different uppercase letter above bars indicates significant differences among soil fractions across all CC treatments at *P* < 0.05. FT, functional type; SF, soil fraction; FT * SF, interaction effect of functional type and soil fraction.


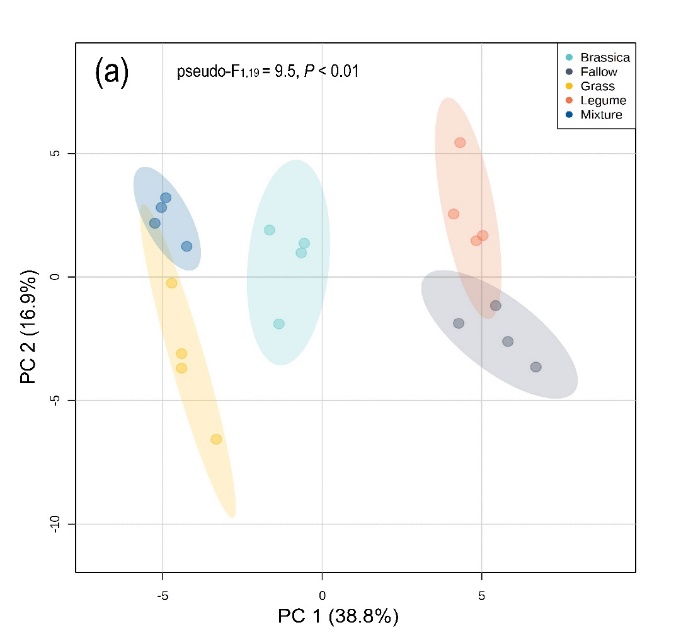

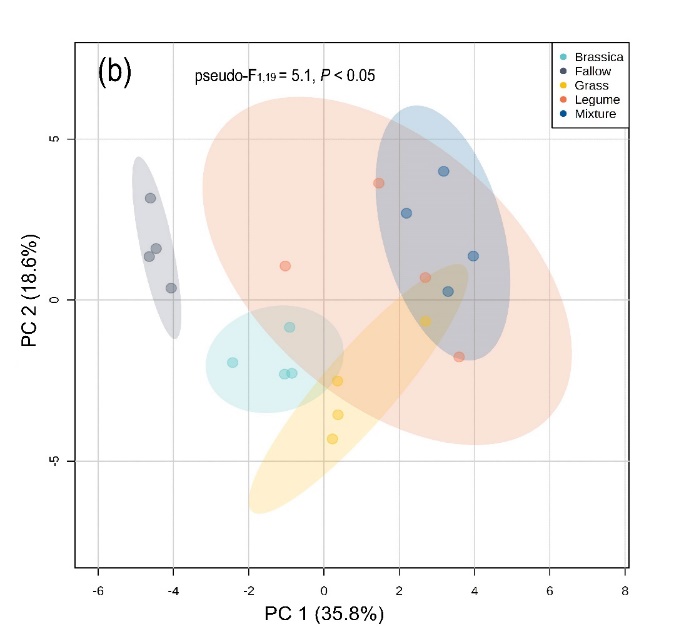


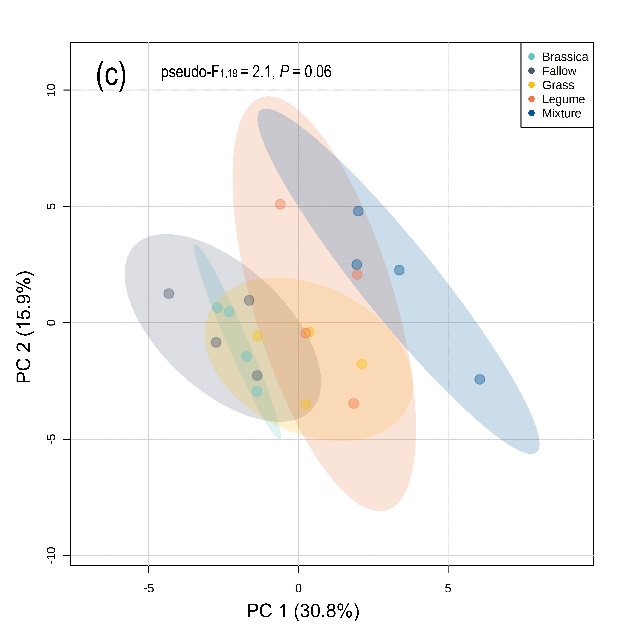


**Fig. S4.** Principal component analysis (PCA) of the concentrations of all identified soil biomarkers in (a) oPOM, (b) MAOM, and (c) fPOM of soils under different CC treatments, with PERMANOVA confirming a significant or marginally significant difference between the groups (pseudo-F_1,19_ = 9.5, *P* < 0.01 for oPOM; pseudo-F_1,19_ = 5.1, *P* < 0.05 for MAOM; pseudo-F_1,19_ = 2.1, *P* = 0.06). Ellipses show clustering of samples by CC treatment.


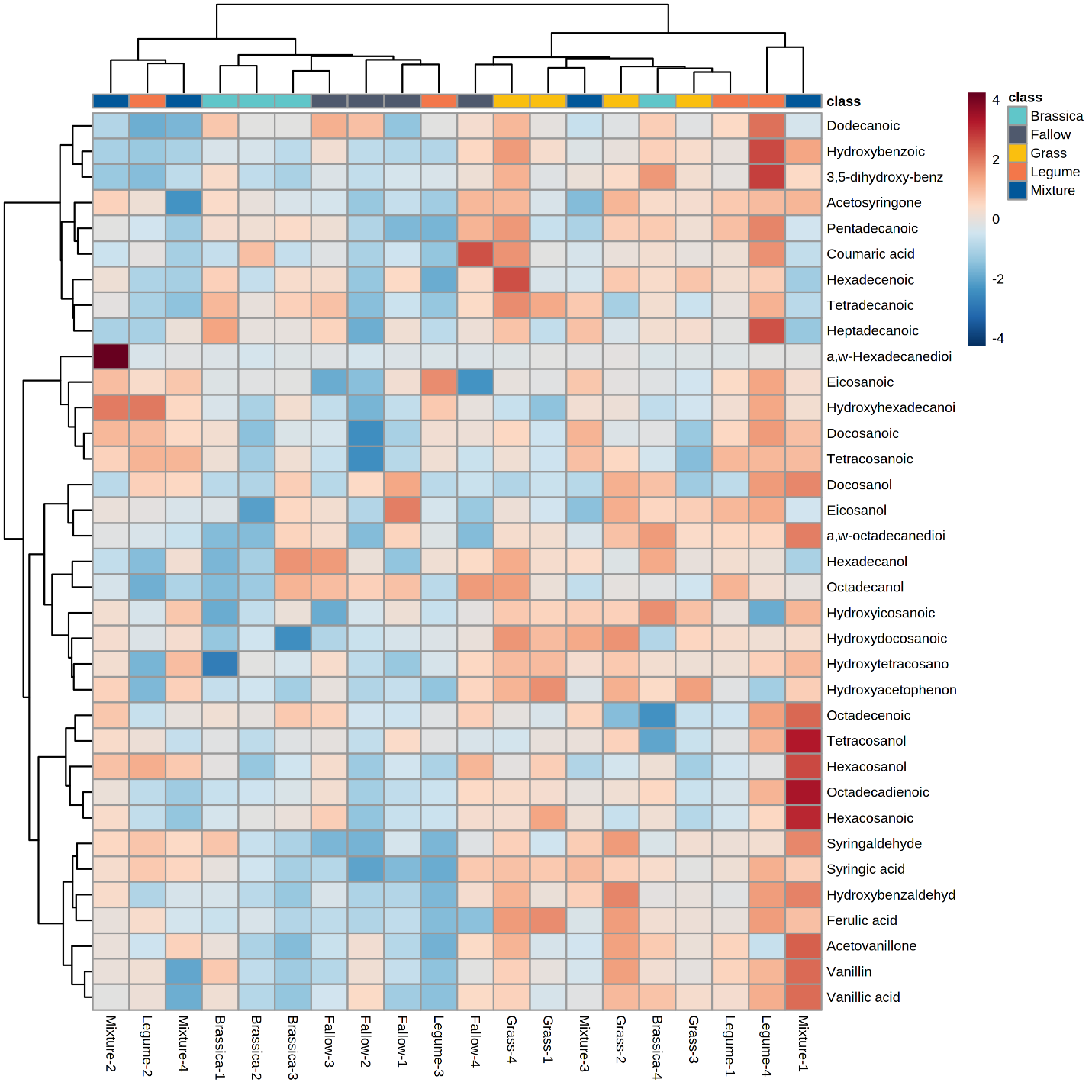


**Fig. S5.** Heatmap and two-way hierarchical clustering of the concentrations of all identified soil biomarkers in fPOM of soils under different CC treatments. Each column represents a sample from a specific CC treatment, and each row represents a positively identified soil biomarker based on mass spectrum information. The red and blue colors in each cell indicate relatively higher and lower concentrations of specific biomarkers in each sample, respectively.

**Table S1.** Average proportion of biomass of different CC functional types (including weeds) in the 5Spp. Mix (Mixture) treatment over the seven-year organic grain rotation

|  | Cover crop functional types | | |  |  |
| --- | --- | --- | --- | --- | --- |
|  | Legume (%) | Brassica (%) | Grass (%) |  | Weeds (%) |
| Above ground biomass | 19.24 | 37.15 | 41.04 |  | 2.56 |
| Below ground biomass | 15.69 | 35.55 | 46.68 |  | 2.08 |

| Cover crop preceding maize | Cumulative soil inorganic N (mg N kg soil^-1^) |
| --- | --- |
| Fallow | 593^b^ |
| **Legume** | **796^a^** |
| Grass | 652^b^ |
| Brassica | 572^b^ |
| Mixture | 631^b^ |

**Table S2**. Cumulative surface soil inorganic N concentrations in different CC treatment during the maize growing season, which could indicate the N supply from cover crops

Different lowercase letter within a column indicates significant differences among CC treatments at *P* < 0.05.

**Table S3.** Seedling rates of different CC treatments

|  |  |  | **No. of Species** | **Crimson clover** | **Austrian winter pea** | **Triticale** | **Canola** | **Red clover** |
| --- | --- | --- | --- | --- | --- | --- | --- | --- |
| **Functional type** | **CC treatment** | **Species composition** |  | **——————— live seeds m^-2^ ———————** | | | | |
| Grass | Triticale | Triticale | 1 |  |  | 300 |  |  |
| Brassica | Canola | Canola | 1 |  |  |  | 400 |  |
| Legume | Crimson clover | Crimson clover | 1 | 600 |  |  |  |  |
| Mixture | 5Spp. Mix | Triticale, Canola, Crimson clover, Austrian Winter pea, Red clover | 5 | 132 | 13.2 | 57 | 60 | 132 |
